# Human Biomonitoring of T-2 Toxin, T-2 Toxin-3-Glucoside and Their Metabolites in Urine through High-Resolution Mass Spectrometry

**DOI:** 10.3390/toxins13120869

**Published:** 2021-12-05

**Authors:** Alfonso Narváez, Luana Izzo, Noelia Pallarés, Luigi Castaldo, Yelko Rodríguez-Carrasco, Alberto Ritieni

**Affiliations:** 1 Department of Pharmacy, Faculty of Pharmacy, University of Naples “Federico II”, Via Domenico Montesano 49, 80131 Naples, Italy; alfonso.narvaezsimon@unina.it (A.N.); luana.izzo@unina.it (L.I.); luigicastaldo1985@yahoo.it (L.C.); alberto.ritieni@unina.it (A.R.); 2 Laboratory of Food Chemistry and Toxicology, Faculty of Pharmacy, University of Valencia, Av. Vicent Andrés Estellés s/n, 46100 Burjassot, Spain; noelia.pallares@uv.es

**Keywords:** human biomonitoring, biomarkers, metabolites, high-resolution mass spectrometry, urine, T-2 toxin, exposure

## Abstract

The metabolic profile of T-2 toxin (T-2) and its modified form T-2-3-glucoside (T-2-3-Glc) remain unexplored in human samples. Therefore, the present study aimed to investigate the presence of T-2, T-2-3-Glc and their respective major metabolites in human urine samples (*n =* 300) collected in South Italy through an ultra-high performance liquid chromatography (UHPLC) coupled to Q-Orbitrap-HRMS methodology. T-2 was quantified in 21% of samples at a mean concentration of 1.34 ng/mg Crea (range: 0.22–6.54 ng/mg Crea). Almost all the major T-2 metabolites previously characterized in vitro were tentatively found, remarking the occurrence of 3′-OH-T-2 (99.7%), T-2 triol (56%) and HT-2 (30%). Regarding T-2-3-Glc, a low prevalence of the parent mycotoxin (1%) and its metabolites were observed, with HT-2-3-Glc (17%) being the most prevalent compound, although hydroxylated products were also detected. Attending to the large number of testing positive for T-2 or its metabolites, this study found a frequent exposure in Italian population.

## 1. Introduction

Mycotoxins are toxic metabolites resultant from the secondary metabolism of several species belonging to the *Fusarium*, *Aspergillus*, *Penicillium*, *Claviceps* and *Alternaria* genera. Under certain conditions, these fungi can colonize a broad variety of crops, eventually leading to the accumulation of mycotoxins. Among the mentioned fungal genera, the *Fusarium* species represents the major mycotoxin-producing pathogens of warm areas from America, Europe and Asia, especially affecting cereal grains and their derived products [1]. Throughout the consumption of contaminated materials, mycotoxins can cause severe adverse health effects in humans, such as immunotoxic, neurotoxic or even carcinogenic effects [2]. Therefore, regulatory authorities have established maximum limits (MLs) in susceptible foods and foodstuffs alongside tolerable daily intake (TDI) values for certain mycotoxins, setting a maximum level of dietary exposure to avoid the appearance of toxic effects.

The traditional methods for estimating dietary exposure are mainly based on the combination of consumption surveys and occurrence data throughout total diet studies or meta-analysis approaches [3,4]. Nevertheless, the outcomes can be considerably biased due to interindividual differences in consumption patterns or to assuming an homogeneous contamination within the same food category. In order to obtain a more reliable estimation of the exposure, human biomonitoring (HBM) studies represent an ideal alternative [5]. These studies involve the measurement of parent mycotoxins and/or their respective metabolites in biological samples, preferably urine, considering its easy and non-invasive collection. In this line, a considerable amount of literature about HBM studies of *Fusarium* toxins and their metabolites occurring in human urine is available, including studies on deoxynivalenol (DON), nivalenol (NIV), fumonisins B1 and B2 (FB1 and FB2), zearalenone (ZEN), enniatin B (ENB) and B1 (ENB1) [6,7,8,9,10]. Nevertheless, the extensive metabolism of T-2 toxin (T-2), a major *Fusarium* toxin included in group 3 of the International Agency for Cancer Research (IARC), has been scarcely studied in biological samples.

T-2 is a type-A trichothecene containing an epoxy group between C12 and C13, a double bond between C9 and C10, and variable acetoxy groups. This toxin mainly occurs in wheat, maize and oat, although it can be found in other cereal grains and derived commodities [11]. Once ingested, T-2 can represent a health concern. T-2 is a major cause of alimentary toxic aleukia, affecting the mucosa and immune system [12]. At the cellular level, T-2 binds to the 60S ribosomal subunit, inhibiting protein synthesis. In addition, T-2 causes oxidative stress, impairs the mitochondrial function by altering the electron transport chain and stimulates apoptosis after activating several MAPK and caspases [13,14,15]. Nevertheless, the toxicity of T-2 highly depends on its metabolites, considering the intensive and rapid metabolization reported after in vitro assays with human liver microsomes. In this line, numerous metabolic pathways were described by Yang et al. [16] for the biotransformation of T-2 into its major metabolic products: hydrolysis for the production of HT-2 toxin (HT-2) and neosolaniol (NEO); hydroxylation, generating 3′-hydroxy-T-2 (3′-OH-T-2) and 3-hydroxy-HT-2 (3′-OH-HT-2, also known as T-2 triol); and glucuronidation, for the production of T-2-3-glucuronic acid (T-2-3-GlcA), HT-2-3-glucuronic acid (HT-2-3-GlcA) and HT-2-4-glucuronic acid (HT-2-4-GlcA).

In addition, T-2 can also be modified in plants by the addition of polar molecules, such as glucose, producing T-2-3-glucoside (T-2-3-Glc) as a result [17]. This modified form has also been repeatedly reported in cereal grains [18]. Therefore, humans could also be exposed to this T-2 modified form through dietary intake. Exposure to T-2-3-Glc represents a health concern, as it can be deconjugated into its free parent toxin within the intestinal tract. However, other metabolites were recently characterized by Yang et al. after in vitro assays with human liver microsomes [19]. Among them, the major metabolites were the hydrolyzed forms HT-2-3-glucoside (HT-2-3-Glc) and NEO-3-glucoside (NEO-3-Glc), and the hydroxylated 3′-hydroxy-T-2-3-Glc (3′-OH-T-2-3-Glc) and 4′-hydroxy-T-2-3-Glc (4′-OH-T-2-3-Glc).

Although Gerding et al. [20] and, recently, De Ruyck et al. [21] have investigated the presence of HT-2-4-GlcA and T-2 triol, respectively, in human urine samples, the complete metabolic profile of major biotransformation products remains unexplored. To overcome the lack of analytical standards, high-resolution mass spectrometry (HRMS) represents an optimal tool for characterizing the metabolic profiles of these parent toxins that require consideration when conducting exposure assessment studies. Therefore, the present study aimed to investigate the presence of T-2, T-2-3-Glc and their respective major metabolites in human urine samples (*n =* 300) collected in South Italy through an ultra-high performance liquid chromatography (UHPLC) coupled to Q-Orbitrap-HRMS methodology. A combined strategy consisting of targeted quantification of T-2 and HT-2, and suspect screening based on exact mass measurement was applied.

## 2. Results and Discussion

### 2.1. Optimization of Q-Orbitrap HRMS Parameters

**Figure 1:**
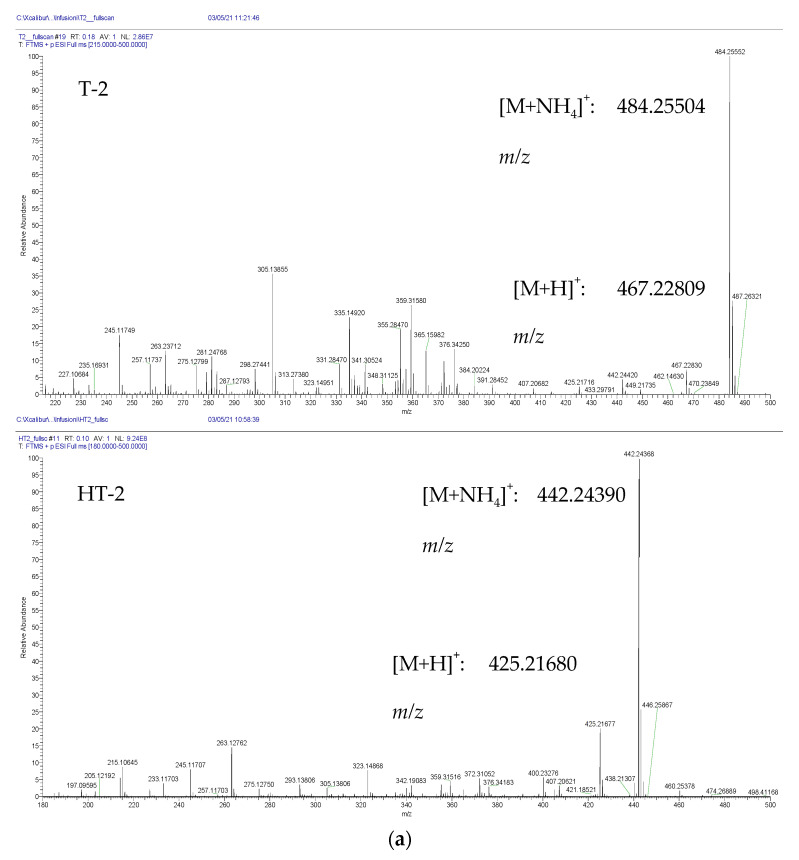

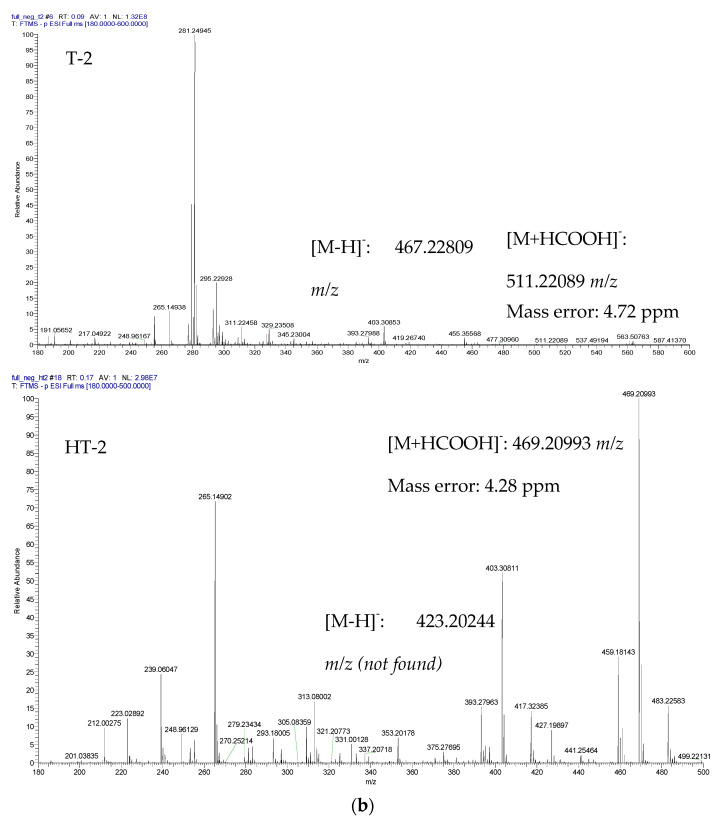
Mass spectra of T-2 and HT-2 in (**a**) positive ionization mode and (**b**) negative ionization mode after a full scan.

Analytical standards of T-2 and HT-2 diluted at 1 µg/mL in methanol were directly infused into the Q-Orbitrap HRMS system at a constant flow rate of 8 µL/min in order to evaluate the MS parameters of both analytes. The system operated in both positive and negative ESI mode to determine the best ionization pattern, and exact mass measurements were compared to the theoretical masses to assess the accuracy, as shown in Figure 1. Both mycotoxins formed stable ammonium adducts in the positive ESI mode with high accuracy (mass error < 2 ppm) that were further used for targeted quantification in the urine samples. Protonated adducts were also observed for both mycotoxins but at a lower relative intensity, whereas negative ionization showed not only lower relative intensity but also lower exact mass accuracy.

Previous in vitro studies with human liver microsomes have tentatively identified the major biotransformation products of T-2 and T-2-3-Glc, and elucidated their structure through mass spectrometry analysis [16,19]. Therefore, the following T-2 metabolites were targeted for retrospective analysis: hydrolysis products (NEO and 4-deAc-NEO), hydroxylation products (3′-OH-T-2 and T-2 triol) and phase II metabolites (T-2-3-GlcA, HT-2-3-GlcA and HT-2-4-GlcA). In addition, the modified form T-2-3-Glc was also included alongside its hydrolyzed (NEO-3-Glc and HT-2-3-Glc) and hydroxylated (3′-OH-T-2-3-Glc and 4′-OH-T-2-3-Glc) metabolic products. Although the biotransformation of T-2-3-Glc could also originate T-2 and its corresponding products, only the glucoside forms were included as T-2-3-Glc metabolites for classification purposes. Tentative identification was performed following a suspect screening approach based on the exact mass measurements. Both the protonated and ammonium adducts of each analyte were initially targeted to select the fragment with the higher intensity for further analysis. In addition, the order of elution was compared to previous methodologies that used reverse-phase chromatography, considering that a more polar character implies a sooner elution, while also allowing a proper differentiation of the isomeric forms. A stringent mass error of < 2 ppm was also set, aiming for a more accurate identification in urine samples. Data for the retention times, theoretical mass, observed mass and mass error for all the assayed compounds are shown in Table 1.

### 2.2. Method Validation

The proposed methodology for the analysis of urine samples was validated in-house according to the in-force legislation [22]. The results from validation experiments are shown in Table 2. The calibration curves built in neat solvent and blank matrix showed good linearity (r^2^ > 0.990) throughout the assayed range of concentrations (20–0.1 ng/mL). After comparing both slopes of the calibration curves, a negligible matrix interference was detected (98–102%). Therefore, the external calibration based on the neat solvent curve was used for quantification purposes. The spiking experiments determined a proper recovery of analytes that ranged from 79% to 116% for the selected fortification levels (5, 1 and 0.5 ng/mL). The maximum relative standard deviations of 19% and 17% after intraday (RSD_r_) and interday (RSD_R_) precision studies were respectively obtained. The absence of coelutant peaks in the matrix after the analysis of blank samples (*n =* 10) confirmed the selectivity of the proposed methodology. LOQs corresponding to T-2 and HT-2 were set at 0.2 ng/mL and 0.4 ng/mL, respectively. Therefore, the proposed methodology fulfilled the validation criteria and was further applied for quantification of T-2 and HT-2 in human urine samples.

Several methodologies have been validated for the quantification of T-2 and HT-2 in urine samples, as shown in Table 3. Liquid chromatography coupled to tandem low-resolution mass spectrometry appears to be the gold standard, although gas chromatography has also been used. Similar to the present study, the use a Q-Orbitrap mass spectrometer in the recent study conducted by Ndaw et al. [23], which remarks the rising use of HRMS techniques in HBM studies, must be highlighted. Apart from quantification, HRMS allows retrospective data analysis for untargeted compounds and suspect screenings. Therefore, HRMS could represent the main tool for future HBM studies considering the complex metabolome occurring in biological samples [24]. In terms of sensitivity, most of the available analytical techniques have reported LOQs ≤ 1 ng/mL for T-2, whereas sensitivity to HT-2 seems to be critically impacted not by the analytical technique but the sample treatment. In this line, direct approaches, such as dilute and shoot or filter and shoot, have shown a strong variability in sensitivity between both analytes. Although these procedures represent a good alternative in HBM studies, based on an easy and quick workflow, these are unable to selectively remove any interference present in the matrix, thus reducing the performance of the methodology. Other sample treatments including a clean-up step, such as QuEChERS or SALLE procedures, obtained better sensitivities for HT-2 when compared to direct approaches. The here-presented methodology, based on a simple SALLE + clean-up procedure and later UHPLC-HR-Q-Orbitrap-MS/MS, showed suitable sensitivity for detecting both analytes at low ng/mL levels.

### 2.3. Urinary Levels of T-2 and HT-2

The validated methodology was applied to 300 human urine samples to assess the urinary levels of T-2 and HT-2. Table 4 reviews previous studies with positive urine samples for any of both mycotoxins alongside the here-obtained results. In the present study, T-2 was quantified in 21% of samples at a mean concentration of 1.34 ng/mg Crea (range: 0.22–6.54 ng/mg Crea), whereas HT-2 showed a slightly higher prevalence, occurring in 30% of samples at a similar mean concentration of 1.23 ng/mg Crea. No significant differences were observed when comparing the concentration levels of both analytes throughout the whole dataset. Nevertheless, a statistically significant positive association between the occurrence of T-2 and HT-2 within the same sample was revealed (*p*-value < 0.01, phi coefficient = 0.479). This co-exposure might be explained by the correlated occurrence of both toxins in foodstuffs, which has been extensively reported in the literature [11,35,36]. Across age and gender groups, both mycotoxins reflected similar distributions in terms of prevalence and concentration, supported by statistical analysis which determined non-significant differences. 

Scarce literature about T-2 or HT-2 occurring in urine samples is available. In addition, most of the published studies have focused on specific cohorts, compiling several criteria in terms of age range, occupational exposure or diseases potentially related to T-2 exposure, as shown in Table 4. In general, a low rate of positive samples has been reported when considering healthy cohorts, with T-2 occurring in a higher number of samples than HT-2 (T-2 < 21.8%; HT-2 < 13.6%). Quantitatively, HT-2 seemed to occur at higher concentrations when compared to T-2 (T-2 < 1.75 ng/mg Crea; HT-2 < 16.81 ng/mg Crea). In contrast with these previous studies, the here-presented results demonstrated a higher prevalence rate for both mycotoxins but at lower concentration values. These inconsistencies in urinary levels could be due to several factors. First, the analytical performance of the abovementioned studies displayed differences in terms of sensitivity for each mycotoxin, with LOQs being 3–8-times higher for HT-2. This could directly translate into lower rates of positive samples and higher mean concentration values. Second, both mycotoxins can be found in foods and foodstuffs, so variable levels can be expected according to dietary habits. In this line, the latest data on food supply quantity by the Food and Agricultural Organization of the United Nations (FAO) presented a higher consumption of cereals and cereal-based products in Italy (160.97 kg/capita/year) when compared to Germany (115.03 kg/capita/year), the United Kingdom (126.75 kg/capita/year) Belgium, the Czech Republic, the Netherlands and Norway (<102.12 kg/capita/year) [37], so higher prevalence could also be expected in the present study. In addition, the results obtained in HBM studies that have focused on a specific age segment (see Table 4, Gratz et al. [32], 2–6 years old; Gerding et al. [20], 20–30 years old) may not be comparable to studies with larger sample sizes due to the potential bias generated by age-related consumption patterns. Last, although little is known about toxicokinetics of T-2 and HT-2 in human, in vivo studies with rats and pigs have reported low half-life values for both toxins in urine, as reviewed by Schelstraete et al. [38], so this might hamper the understanding of urinary levels in human samples too. Therefore, these results provide even more evidence on the occurrence of T-2 in human urine and support the development of more sensitive analytical techniques for its application in HBM studies to clarify the impact of T-2 in humans.

### 2.4. Retrospective Analysis of Urine Samples

**Figure 2:**
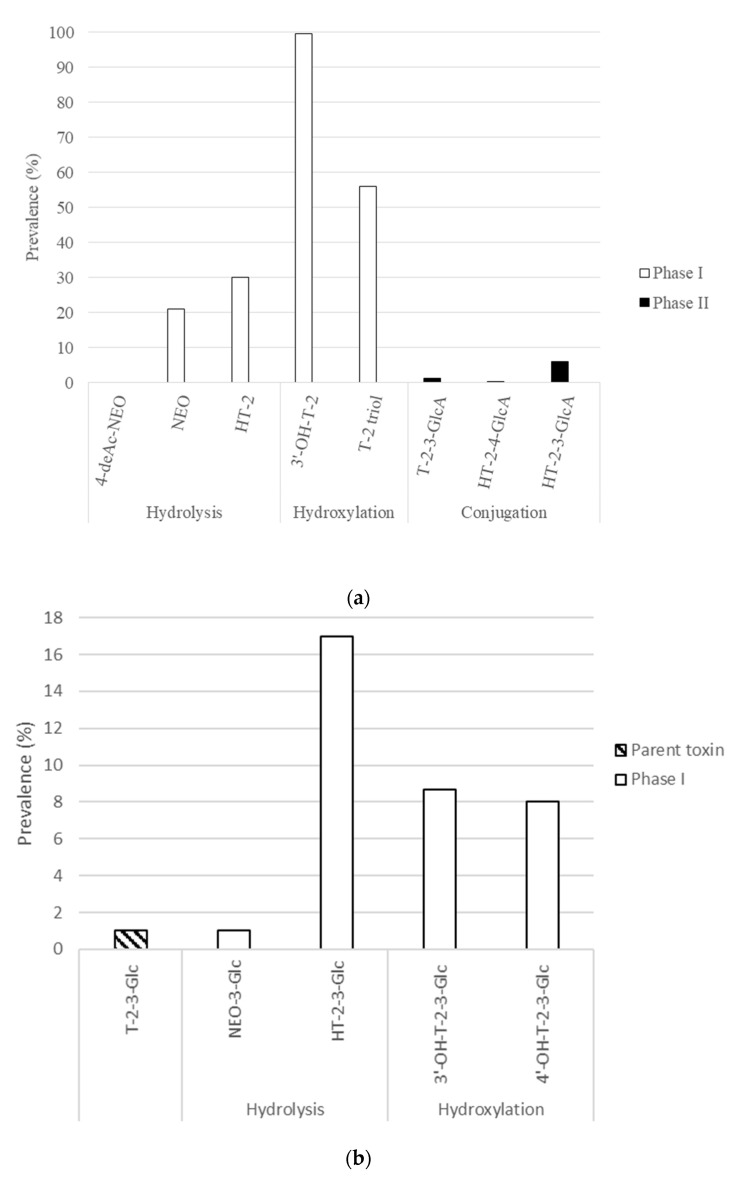
Prevalence of (**a**) T-2 metabolites and (**b**) T-2-3-Glc and its phase I metabolites in 300 human urine samples. Although HT-2 was assessed through a targeted methodology, it was introduced in this section as a major T-2 metabolite.

Data collected after UHPLC-Q-Orbitrap-HRMS analysis were manually examined in Xcalibur Qual Browser v.3.1.66 to evaluate the tentative presence of NEO, 4-deAc-NEO, 3′-OH-T-2, T-2, T-2-3-GlcA, HT-2-3-GlcA, HT-2-4-GlcA, T-2-3-Glc, NEO-3-Glc, HT-2-3-Glc, 3′-OH-T-2-3-Glc and 4′-OH-T-2-3-Glc in 300 human urine samples. Identification was based on exact mass identification in at least two of the three replicates, with a stringent mass error of 2 ppm using the molecular formulas previously reported in the literature [16,19], thus corresponding to a level 5 of certainty as established by Schymanski et al. [39]. The results are shown in Figure 2.

Almost all the major T-2 metabolites previously characterized in vitro were tentatively found in human urine within the following order of prevalence: 3′-OH-T-2 (99.7%) > T-2 triol (56%) > HT-2 (30%) > NEO (21%) > HT-2-3-GlcA (6%) > T-2-3-GlcA (1.3%) > HT-2-4-GlcA (0.3%) > 4-deAc-NEO (0%). These results could indicate phase I metabolism as the preferential biotransformation pathway, as evidenced by the high proportion of 3′-OH-T-2 and T-2 triol and the low relevance of conjugated metabolites, although dietary exposure could also take part on these outcomes. Furthermore, among phase I metabolites, statistical analysis revealed a significantly higher frequency of hydroxylated metabolites when compared to the hydrolyzed products (*p*-value < 0.01). Therefore, hydroxylation seemed to be the main biotransformation pathway of T-2. Although the presence of HT-2 and NEO could indicate hydrolysis as an alternative reaction, their prevalence could be partly due to dietary exposure considering that they extensively occur in foodstuffs [40]. The low relevance of conjugated metabolites in human urine has been previously reported by Gerding et al. [20], who did not find any positive samples for HT-2-4-GlcA.

These results are in contrast with the human in vitro assays conducted by Yang et al. [16], which found HT-2 as the most predominant metabolites, whereas T-2 triol was the preferred hydroxylation product. Nevertheless, different metabolic profiles have been observed after comparing the in vivo and in vitro data from other species. Similar to human in vitro data, other previous assays with liver microsomes of chickens and rats have revealed a predominance of hydrolyzed metabolites, whereas in vivo experiments in both species have remarked hydroxylation as the main biotransformation pathway and did not observe conjugation [16,41], in line with the here-observed findings. The high relevance of hydroxylated compounds, especially 3′-OH-T-2, could represent a concern considering that it does not exert a significantly lower toxicity. Moreover, 3′-OH-T-2 might even display a faintly higher toxicity when compared to its parent mycotoxin [42,43].

Regarding the T-2-3-Glc metabolic profile, a low prevalence of the parent mycotoxin (1%) and its metabolites was observed: HT-2-3-Glc (17%) > 3′-OH-T-2-3-Glc (8.7%) > 4′-OH-T-2-3-Glc (8%) > NEO-3-Glc (1%). The observed low urinary prevalence is in agreement with findings reported by Yang et al. [19], who observed that T-2-3-Glc and its metabolites were mainly excreted in feces, whereas only traces were observed in urine after the oral administration of T-2-3-Glc to rats. Nevertheless, only T-2, HT-2 and 3′-OH-T-2-3-Glc were reported in urine, whereas the here-obtained results highlighted HT-2-3-Glc as the major T-2-3-Glc metabolite, being tentatively detected at a significantly higher frequency when compared to the rest of products (*p*-value < 0.01) This discrepancy could be addressed by considering not only in vitro/in vivo and interspecies differences but also potential dietary exposure. Although the presence of HT-2-3-Glc has been scarcely studied in foodstuffs, the available studies have reported a high prevalence in oats, wheat and barley samples [44].

The vast occurrence of metabolites in human urine samples indicated an extensive biotransformation of T-2 and T-2-3-Glc. Although scarce information is available on in vivo metabolism, a similar pattern across species has been observed with a preferential metabolism through phase I reactions for T-2 and low-to-no relevance of conjugation reactions.

## 3. Conclusions

A simple SALLE procedure followed by a UHPLC-Q-Orbitrap HRMS methodology was applied to monitor T-2, T-2-3-Glc and their corresponding metabolites in 300 urine samples collected from volunteers in South Italy. After validation of the analytical procedure according to the current legislation, a combined approach was successfully used for the quantification of T-2 and HT-2, and a suspect screening for the tentative identification of the major T-2 and T-2-3-Glc metabolites.

The metabolic profile of T-2 in urine samples indicated hydroxylation as the main biotransformation pathway, with T-2 triol and 3′-OH-T-2 occurring at significantly higher prevalence when compared to the rest of metabolites, whereas conjugation reactions appeared to be residual in line with in vivo findings from other species. The hydroxylated metabolite 3′-OH-T-2 was tentatively found in almost all samples, which could represent a concern considering that it does not show significantly lower toxicity compared to its parent mycotoxin. The modified form T-2-3-Glc and its corresponding metabolites were less frequent in urine samples, in accordance with other previous in vivo studies that observed an almost complete excretion in feces and only determined traces of metabolites in urine. Similarly, hydroxylated products 3′-OH-T-2-3-Glc and 4′-OH-T-2-3-Glc were tentatively identified. Therefore, the observed urinary metabolic profile of T-2 and T-2-3-Glc reveal similarities with other species.

Attending to the large number of samples testing positive for T-2 or its metabolites, this study also found a frequent exposure to T-2, although there is a considerable variability in the available HMB studies that have used urine samples. Thus, more sensitive analytical techniques should be validated for their application in biological matrices in order to clarify the impact of T-2 in humans. In addition, considering the frequent exposure to several metabolites that can also occur in foodstuffs, such as T-2 triol or HT-2-3-Glc, analytical methodologies in food analyses should incorporate them. This could help to elucidate whether the presence of these metabolites is due to dietary exposure or to T-2/T-2-3-Glc metabolism.

## 4. Material and Methods

### 4.1. Chemicals, Reagents and Materials

Water for the LC mobile phase (LC-MS grade), acetonitrile (AcN) and methanol (MeOH) were acquired from Merck (Darmstadt, Germany). Formic acid (MS grade) was supplied by Carlo Erba reagents (Cornaredo, Italy). Ammonium formate (analytical grade) was purchased from Fluka (Milan, Italy). Octadecyl carbon chain-bonded silica (C18) (analytical grade) and sodium chloride (NaCl) were obtained from Sigma Aldrich (Milan, Italy). Conical centrifuge polypropylene tubes of 15 mL were provided by BD Falcon (Milan, Italy). Syringe filters with polytetrafluoroethylene membrane (PTFE, 15 mm, diameter 0.2 µm) were acquired from Phenomenex (Castel Maggiore, Italy).

Analytical standards of T-2 and HT-2 (HPLC purity > 98%) were supplied by from Sigma-Aldrich (Milan, Italy). Stock solutions were built by diluting 1 mg of each standard mycotoxin in 1 mL of MeOH. Then, working solutions were prepared by properly diluting with MeOH/H_2_O (70:30 *v*/*v*) 0.1% formic acid to reach the concentrations needed for spiking experiments (5, 1 and 0.5 ng/g). The solutions were kept in securely closed vials at −20 °C.

### 4.2. Sampling

First-spot morning urine (50 mL) samples from 300 volunteers were collected during January and February 2018 in the Campania region (Southern Italy) and stored in sterile plastic vessels. Samples were aliquoted and storage at −20 °C until further analysis to avoid stability issues. Volunteers were selected among students, academic and non-academic staff of the Faculty of Pharmacy of University of Naples “Federico II” considering the following exclusion criteria: (i) susceptible people to occupational exposure, such as veterinarians and farmers, were excluded; (ii) only one member per family was allowed; (iii) people with serious bile, kidney or liver problems were not eligible due to potential interferences with the metabolism of mycotoxins. No diet limitations were established during the sampling. All participants provided written consent in accordance with the Helsinki Declaration on ethical principles for medical research involving human subjects. This project was approved by the University of Valencia Ethics Committee. The sample size (*n =* 300) selected is consistent with previous HBM studies of food contaminants [5].

Participants were asked to specify their age and gender in the vessel for further data treatment. The sampling attempted to maintain the gender parity (male: 45.7%, female: 54.3%). Three age groups were considered: ≥60 years old (*n =* 134), from 31 to 59 years old (*n =* 72) and ≤31 years old (*n =* 94). Samples with undetectable levels of mycotoxins were used for recovery studies.

### 4.3. Extraction Proczedure

Sample preparation procedure was conducted following a methodology previously developed by Rodríguez-Carrasco et al. [8]. Briefly, 1.5 mL of urine sample was transferred into a 2 mL Eppendorf Safe-Lock Microcentrifuge tube and centrifuged at 3926× *g* for 3 min. Afterward, 1 mL of the supernatant was collected and placed into a 15 mL screw cap test tube with conical bottom alongside 1 mL of acetonitrile. The mixture was vortexed for 30 s. Then, a blend of 30 mg of C18 sorbent and 0.3 g of NaCl were added and vortexed for 30 s and centrifuged at 3926× *g* at 4 °C for 3 min. Finally, the upper layer was transferred into another 15 mL screw cap test tube with conical bottom and evaporated to dryness under nitrogen flow at 45 °C, reconstituted with 0.5 mL of MeOH/H_2_O (70:30 *v/v*) 0.1% formic acid and filtered through a 0.2 µm filter prior to UHPLC-Q-Orbitrap HRMS analysis.

### 4.4. UHPLC-Q-Orbitrap HRMS Analysis

Chromatographic separation through UHPLC was conducted using a Dionex Ultimate 3000 (Thermo Fisher Scientific, Waltham, MA, USA) instrument equipped with a degassing system, an auto sampler device, a quaternary UHPLC pump working at 1250 bar and a thermostated (30 °C) Luna Omega column (50 × 2.1 mm, 1.6 µm, Phenomenex). As mobile phases, water (A) and methanol (B), both containing 0.1% formic acid and 5 mM ammonium formate, were used. The chromatographic gradient followed the next configuration: initial 0% of phase B was kept for 1 min, then increased to 95% in 1 min and held for 0.5 min. Next, the gradient went back to 75% of B in 2.5 min and then decreased again until 60% in 1 min. Finally, the gradient switched back to 0% of B in 0.5 min and was held during 1.5 min for column re-equilibration, accounting for an entire run time of 8 min. The flow rate was set at 0.4 mL/min, and an aliquot of 5 µL of sample was injected.

After chromatographic separation, samples were assessed through HRMS using a Q-Exactive Orbitrap system. The analysis was conducted in positive electrospray ionization (ESI) and full scan mode. The ionization parameters were: spray voltage, 4 kV; capillary temperature, 290 °C; auxiliary gas (N2 > 95%), 10; auxiliary gas heater temperature, 305 °C; sheath gas pressure (N2 > 95%), 35; S-lens radio frequency (RF) level, 50. Full scan data collection was performed with the following settings: resolving power 70,000 full width at half maximum (FWHM) at 200 *m*/*z*, automatic gain control (AGC) target 1 × 106, injection time 200 ms, scan range from 100 to 800 *m*/*z*, scan rate 2 scans/s. A stringent mass tolerance of 2 ppm was set for identification. Data analysis was performed using Quan/Qual Browser Xcalibur v.3.1.66 (Thermo Fisher Scientific, Waltham, MA, USA).

### 4.5. Method Validation

In-house validation was conducted following the in-force legislation [22] in terms of linearity, matrix-induced deviations, selectivity, trueness, within-laboratory reproducibility, repeatability and limits of quantification (LOQs). Linearity (r2) was obtained after building the neat solvent and matrix-matched calibration curves using T-2 and HT-2 analytical standards. Concentrations ranged from 20 to 0.1 ng/mL, with each level of the calibration curves showing a relative standard deviation (RSD) < 20% compared to the theoretical concentration. The comparison of both calibration curves throughout their corresponding slopes allowed the assessment of the signal suppression/enhancement effect (%SSE), following the next equation:

%SSE = Sm/Ss × 100(1)

where Sm is the matrix-matched calibration slope and Ss is the solvent calibration slope. An %SSE below 100% was translated into signal suppression, whereas values above 100% represented signal enhancement in the range of concentrations assayed. Trueness was evaluated through recovery experiments, spiking known blank samples at three different levels (5, 1 and 0.5 ng/mL). Experiments were conducted in triplicate on three nonconsecutive days and reflected as interday (within-laboratory reproducibility, RSDR) or intraday (repeatability, RSDr) relative standard deviation. Selectivity was assessed to determine the potential presence of coelutants in the matrix. Thus, blank samples (*n =* 10) were injected immediately after the highest calibration sample. For confirmation criteria, the retention times of the analytes in standards and samples were compared. LOQs were considered as the lowest concentration where the molecular ion could be identified inside the linear range, considering a mass error below 5 ppm.

### 4.6. Quality Control/Quality Assurance

Spectral and chromatographic data were combined for the correct identification of the analytes. Retention times attached to the assayed analytes were compared in both positive samples and standards in neat solvent at a tolerance of ±2.5% of the total run time (8 min). Data quality was verified through the inclusion of a comprehensive range of quality assurance and quality control procedures. Each batch of samples contained a reagent blank, a procedural blank and a matrix-matched calibration in order to evaluate the robustness and stability of the system throughout the whole analysis.

### 4.7. Creatinine Analysis

Urinary levels of creatinine were calculated throughout a spectrophotometric assay previously performed by Rodríguez-Carrasco et al. [23]. In brief, 1000 mM NaOH was mixed with 3.5 mM picric acid to obtain alkaline picrate. The solution was stored in dark conditions in an amber glass container. Urine samples were then diluted using ultrapure water (1:10 *v*/*v*) and 1 mL was reacted with 1 mL of alkaline picrate solution. The optical density was measured after 30 min at 500 nm using a Shimadzu mini 1240 spectrophotometer. Finally, concentrations of mycotoxins were related to the creatinine content of the corresponding sample and expressed as ng/mg Crea.

### 4.8. Statistical Analysis

Statistical data treatment was carried out in software package IBM SPSS v.25. The Mann–Whitney U test was used to detect quantitative differences between T-2 and HT-2 in the assayed samples according to gender and age groups. Categorical data, as the prevalence of T-2, T-2-3-Glc and its corresponding metabolites across gender and age groups, were compared throughout the Pearson chi-square tests. A confidence level of 95% was selected for data treatment, and a *p*-value < 0.01 was considered as significant.

## Figures and Tables

**Table 1:** UHPLC-Q-Orbitrap-HRMS parameters for the targeted and non-targeted analytes.

Analyte	Retention Time (min)	Molecular Formula	Adduct Ion	Exact Mass (*m*/*z*)	Observed Mass (*m*/*z*)	Mass Error (ppm)
Parent mycotoxin						
T-2	4.82	C_24_H_34_O_9_	[M + NH_4_]^+^	484.25411	484.25504	1.92
Phase I metabolites—Hydrolyzed group						
4-deAc-NEO ^a^	-	C_17_H_24_O_7_	[M + NH_4_]^+^	358.18602	-	-
			[M + H]^+^	341.15950	-	-
HT-2	4.79	C_22_H_32_O_8_	[M + NH_4_]^+^	442.24354	442.24390	0.81
NEO	4.25	C_19_H_26_O_8_	[M + NH_4_]^+^	400.19669	400.19659	−0.25
Phase I metabolites—Hydroxylated group						
T-2 triol	4.7	C_20_H_30_O_7_	[M + H]^+^	383.20642	383.20662	0.52
3′-OH-T-2	4.78	C_24_H_34_O_10_	[M + NH_4_]^+^	500.24902	500.24926	0.48
Phase II metabolites—Conjugated group						
T-2-3-GlcA	4.69	C_30_H_42_O_15_	[M + H]^+^	643.25964	643.25974	0.16
HT-2-3-GlcA	4.67	C_28_H_40_O_14_	[M + H]^+^	601.24908	601.24998	1.5
HT-2-4-GlcA	4.39	C_28_H_40_O_14_	[M + H]^+^	601.24908	601.24998	1.5
						
Parent mycotoxin						
T-2-3-Glc	4.38	C_30_H_44_O_14_	[M + NH_4_]^+^	646.30693	646.30793	1.55
Phase I metabolites—Hydrolyzed group						
HT-2-3-Glc	4.59	C_28_H_42_O_13_	[M + NH_4_]^+^	604.29636	604.29616	−0.33
NEO-3-Glc	3.85	C_25_H_36_O_13_	[M + NH_4_]^+^	562.24941	562.24897	−0.78
Phase I metabolites—Hydroxylated group						
3′-OH-T-2-3-Glc	3.91	C_30_H_44_O_15_	[M + NH_4_]^+^	662.30184	662.30121	−0.95
4′-OH-T-2-3-Glc	4.04	C_30_H_44_O_15_	[M + NH_4_]^+^	662.30184	662.30121	−0.95

^a^ Neither the protonated nor the ammonium adducts were observed.

**Table 2:** Method performance for T-2 and HT-2.

Analyte	Linearity (r^2^)	SSE (%)	Recovery (%)			Precision (%) [RSD_r_, (RSD_R_)]			
			5 ng/mL	1 ng/mL	0.5 ng/mL	5 ng/mL	1 ng/mL	0.5 ng/mL	LOQ (ng/mL)
HT-2	0.9901	102	116	86	85	11 (9)	5 (6)	12 (17)	0.4
T-2	0.9944	98	116	87	79	8 (7)	11 (17)	19 (14)	0.2

**Table 3:** Previous methodologies for the quantification of T-2 and HT-2 in human urine samples.

Sample Treatment	Analytical Method	LOQ ^a^ (ng/mL)		Reference
		T-2	HT-2	
Dilute and shoot	UHPLC-Q-TRAP-MS/MS	0.5	4	Gerding et al. [20]
SALLE	UPLC-QqQ-MS/MS	0.013	0.036	De Ruyck et al. [21]
Clean-up	UHPLC-HR-Q-Orbitrap-MS/MS	1	0.5	Ndaw et al. [23]
LLE	HPLC-Q-TRAP-MS/MS	6.7	67	Abia et al. [25]
QuEChERS	GC-QqQ-MS/MS	1	2	Rodríguez-Carrasco et al. [26]
Dilute and shoot	UHPLC-Q-TRAP-MS/MS	1	40	Ezekiel et al. [27]
LLE	HPLC-Q-TRAP-MS/MS	1	40	Warth et al. [28]
Filter and shoot	UHPLC-QqQ-MS/MS	0.03	0.5	Heyndrickx et al. [29]
LLE	UHPLC-QqQ-MS/MS	0.1	0.84	
LLE	HPLC-Q-TRAP-MS/MS	0.2	9	Gerding et al. [30]
LLE	UHPLC-Q-TRAP-MS/MS	0.1	0.5	Fan et al. [31]
IA-SPE	UHPLC-QqQ-MS/MS	0.013	0.031	Gratz et al. [32]
DLLME	GC-QqQ-MS/MS	1	2	Niknejad et al. [33]
Dilute and shoot	HPLC-Q-TRAP-MS/MS	10	5	Duringer et al. [34]
SALLE + clean-up	UHPLC-HR-Q-Orbitrap-MS/MS	0.2	0.4	Present study

^a^ Values considering the dilution/concentration factor of each methodology; LLE = liquid-liquid extraction; QuEChERS = quick, easy, cheap, effective, rugged and safe; IA = immunoaffinity; SPE = solid-phase extraction; DLLME = dispersive liquid-liquid microextraction; SALLE = salt-assisted liquid-liquid extraction; HPLC = high-performance liquid chromatography; Q = quadrupole; MS/MS = tandem mass spectrometry; GC = gas chromatography; QqQ = triple quadrupole; UHPLC = ultra-high performance liquid chromatography; HR = high resolution.

**Table 4:** Previous human biomonitoring studies with positive urine samples for T-2 and/or HT-2.

Provenance	Cohort (age)	Samples (n)	LOQ (ng/mL)^a^		Prevalence (%)		Range of Concentration (ng/mg Crea)		Mean (ng/mg Crea)		Reference
			T-2	HT-2	T-2	HT-2	T-2	HT-2	T-2	HT-2	
Germany	Adults (20–30)	101	0.5	4	1	nd	<LOQ	nd	na	nd	Gerding et al. [20]
Belgium, Czech Republic, Netherlands, and Norway	Adults (45–65)	188	0.013	0.036	21.8	6.4	<LOQ—0.77	<LOQ—4.60	0.05 ^c,d^	0.48 ^c,d^	De Ruyck et al. [21]
France	Adults, grain elevator workers (19–56)	18	1	0.5	4	4	<LOQ—2.73	<LOQ—3.29	na	na	Ndaw et al. [23]
Spain	Children (8–14)	16			nd	6.2	nd	12.6 ^b^	nd	12.6	Rodríguez-Carrasco et al. [26]
	Young adults (18–28)	16			nd	nd	nd	nd	nd	nd	
	Adults (>28)	22			nd	13.6	nd	15.8 ^b^	nd	14.3	
	Total	54	1	2	nd	7.4	nd	15.8 ^b^	nd	na	
China	Adults (18–66)	260	0.1	0.5	2.3	nd	0.392–4.23	nd	1.75	nd	Fan et al. [31]
United Kingdom	Children (2–6)	21	0.013	0.031	5	5	0.03	6.13	0.03	6.13	Gratz et al. [32]
Iran	Adults, esophageal cancer patients (50–92)	17			6	18	na	na	44.7	29.09	Niknejad et al. [33]
	Adults, control group (20–46)	10	1	2	nd	10	nd	na	nd	16.81	
Uganda	Children, nodding syndrome patients (5–18)	50			74	nd	0–288 ^c^	nd	29 ^c^	nd	Duringer et al. [34]
	Children, control group (5–18)	50			70	nd	0–425 ^c^	nd	49 ^c^	nd	
	Total	100	10	5	72	nd	0–425 ^c^	nd	39 ^c^	nd	
Italy	Children, teenagers and adults (≤30)	94			20	32	0.42–2.37	0.44–2.32	1.26	1.19	Present study
	Adults (31–59)	72			19	28	0.33–6.54	0.46–2.75	1.48	1.48	
	Elderly (≥60)	134			22	30	0.22–2.51	0.44–2.39	1.4	1.13	
	Total	300	0.2	0.4	21	30	0.22–6.54	0.44–2.75	1.34	1.23	

^a^ Values considering dilution/concentration factor of each methodology; ^b^ Only maximum value available; ^c^ Values expressed as ng/mL without applying; creatinine correction; ^d^ Referring to median values instead of mean; nd = not detected; na *=* not available.

## Data Availability

The data presented in this study are available on request from the corresponding author. The data are not publicly available for preserving the privacy of the volunteers that participated in the present study.
